# How the Management of Biochemical Recurrence in Prostate Cancer Will Be Modified by the Concept of Anticipation and Incrementation of Therapy

**DOI:** 10.3390/cancers16040764

**Published:** 2024-02-13

**Authors:** Alessandro Sciarra, Valerio Santarelli, Stefano Salciccia, Martina Moriconi, Greta Basile, Loreonzo Santodirocco, Dalila Carino, Marco Frisenda, Giovanni Di Pierro, Francesco Del Giudice, Alessandro Gentilucci, Giulio Bevilacqua

**Affiliations:** Department Materno Infantile e Scienze Urologiche, Sapienza University, Viale Policlinico 155, 00161 Rome, Italy; va.santarelli@uniroma1.it (V.S.); stefano.salciccia@uniroma1.it (S.S.); martina.moriconi@uniroma1.it (M.M.); greta.basile@uniroma1.it (G.B.); loreonzo.santodirocco@uniroma1.it (L.S.); dalila.carino@uniroma1.it (D.C.); marco.frisenda@uniroma1.it (M.F.); giovanni.dipierro@uniroma1.it (G.D.P.); francesco.delgiudice@uniroma1.it (F.D.G.); alegenti@yahoo.it (A.G.); giulio.bevilacqua@uniroma1.it (G.B.)

**Keywords:** prostatic neoplasm, biochemical recurrence, androgen receptor signaling inhibitors, androgen deprivation therapy

## Abstract

**Simple Summary:**

The risk of early progression to metastatic disease in the event of biochemical recurrence (BCR) after primary treatment for prostate cancer (PC) is extremely variable. PSA-doubling time (DT) is the best parameter currently available to define patients who are at a higher risk of progression, but a single temporal parameter is not sufficient to describe the heterogeneity of this condition. A real precision medicine approach is strongly suggested to offer tailored management to patients with biochemical relapse. At present, patients with low-risk BCR should be offered active observation, while patients with high-risk BCR are likely to benefit from early systemic therapy. Results from the EMBARK study are likely to impact how physicians choose to manage high-risk BCR. The introduction of the concept of anticipation and intensification of treatment through the use of androgen receptor signaling inhibitors (ARSIs) and ADT combination therapy in this indolent but crucial phase is the main innovation of the study. The aim of this review is to provide a comprehensive understanding of the definition, classification, diagnosis, and treatment modalities of BCR, with a focus on the latest innovations and ongoing trials in the management of high-risk BCR.

**Abstract:**

Biochemical recurrence (BCR) after primary treatments for prostate cancer (PC) is an extremely heterogeneous phase and at least a stratification into low- and high-risk cases for early progression in metastatic disease is necessary. At present, PSA-DT represents the best parameter to define low- and high-risk BCR PC, but real precision medicine is strongly suggested to define tailored management for patients with BCR. Before defining management, it is necessary to exclude the presence of low-volume metastasis associated with PSA progression using new-generation imaging, preferably with PSMA PET/CT. Low-risk BCR cases should be actively observed without early systemic therapies. Early treatment of low-risk BCR with continuous androgen deprivation therapy (ADT) can produce disadvantages such as the development of castration resistance before the appearance of metastases (non-metastatic castration-resistant PC). Patients with high-risk BCR benefit from early systemic therapy. Even with overall survival (OS) as the primary treatment endpoint, metastasis-free survival (MFS) should be used as a surrogate endpoint in clinical trials, especially in long survival stages of the disease. The EMBARK study has greatly influenced the management of high-risk BCR, by introducing the concept of anticipation and intensification through the use of androgen receptor signaling inhibitors (ARSIs) and ADT combination therapy. In high-risk (PSA-DT ≤ 9 months) BCR cases, the combination of enzalutamide with leuprolide significantly improves MFS when compared to leuprolide alone, maintaining an unchanged quality of life in the asymptomatic phase of the disease. The possibility of using ARSIs alone in this early disease setting is suggested by the EMBARK study (arm with enzalutamide alone) with less evidence than with the intensification of the combination therapy. Continued use versus discontinuation of enzalutamide plus leuprolide intensified therapy upon reaching undetectable PSA levels needs to be better defined with further analysis. Real-world analysis must verify the significant results obtained in the context of a phase 3 study.

## 1. Introduction

The concept of biochemical recurrence (BCR) in prostate cancer (PC) and the relevance it has in clinical practice is unique if compared with that in other neoplasms. BCR represents, in most cases, the first form of failure after primary treatment with curative intent. It is certainly a concept better defined after radical prostatectomy (RP) but still present even after radiotherapy (RT); it develops in a significant percentage of cases subjected to such treatments and defines their limits in terms of radicality.

In the case of BCR, the patient is informed of the presence of reproducing cancerous cells after an initial silent phase; however, he is an asymptomatic patient in a state of full well-being. The isolated progressive increase in total prostatic specific antigen (PSA) in these patients describes the stage of the disease with completely negative imaging and clinical findings. It is important to remember that these patients are in a condition of complete well-being and that in a non-negligible percentage of cases, they will remain in this condition for a period of more than 5 years, without developing clinical or radiological progression. In these cases, active surveillance without treatment may represent the best choice, often thwarted by a “PSA syndrome” with close checks and initiation of therapies against PSA rather than against PC. On the other hand, a percentage of these BCRs shortly develop clinical and radiological progression with the appearance of distant metastases. The survival and quality of life of a patient with non-metastatic PC is certainly better than that with metastatic PC, and the prevention of the development of a metastatic phase, even if hormone sensitive, is certainly a significant target for these subjects. Consequently, patients with BCR represent an extremely heterogeneous PC category and one of the most complex phases to manage.

The first unmet target is to correctly stratify patients into BCR at low and high risk of early progression to a metastatic disease, identifying the factors useful for this definition. The second target is to free low-risk BCR patients from unnecessary and often harmful treatments as well as from overly burdensome follow-ups. The third target is to prolong as much as possible, through effective treatments, the phase of simple BCR in the high-risk group, preventing and slowing down the progression to the metastatic stage.

BCR represents one of the fields of PC where precision medicine and tailored management based on predictive parameters not limited to PSA is necessary. On the contrary, to date the management of BCR remains extremely heterogeneous in different centers, often submitted to an overtreatment with different strategies.

The concept of anticipation and intensification of therapy, which in recent years has overwhelmed PC starting from the later phases up to globally affecting metastatic hormone sensitive patients, will soon also involve patients with BCR after primary treatment. This aspect makes it even more urgent to correctly stratify and define patients with BCR into low and high risk of early progression to the metastatic phase, so as to precisely identify when anticipation and intensification of treatment may be considered appropriate.

## 2. Definition and Incidence of Biochemical Recurrence after Primary Treatments

### 2.1. Definition and Incidence

RP and RT for PC generally provide pleasing disease control and favorable long-term survival. However, between 27% and 53% of all patients undergoing RP or RT develop PSA recurrence [1]. Approximately 30% (20–40%) of patients following RP and 30–50% of males treated with RT will experience BCR within 10 years post-therapy [2]. For several years, PSA has been used for detection of recurrent and progressive disease after primary treatments. According to the European Association of Urology (EAU) guidelines, a progressive and confirmed serum PSA level > 0.2 ng/mL after RP is considered biochemical recurrence [3]. The use of ultrasensitive PSA assays for routine post-RP follow-up is still debatable. Males who have a PSA nadir of less than 0.01 ng/mL have a high (96%) chance of not having a relapse within two years. Furthermore, clinical features such as ISUP grade and surgical margin status combined with a post-RP PSA levels > 0.01 ng/mL are able to predict BCR and are helpful in determining follow-up intervals [4]. A PSA increase >2 ng/mL higher than the PSA nadir value, regardless of the serum concentration of the nadir, is the RTOG-ASTRO Phoenix Consensus Conference definition of PSA failure following primary RT [5]. The frequent association of androgen deprivation therapy (ADT) with RT in intermediate- and high-risk PC can make the definition and identification of BCR more complex.

### 2.2. How to Define Low- and High-Risk BCR

Over time, it was possible to realize that PSA relapse after primary treatments has different meanings according to the clinicopathological features, such as the Gleason score, PSA doubling time (PSA-DT), clinical stage, and surgical margins’ status (Table 1).

The EAU guidelines recommend the first PSA test to be performed three months after surgery, but PSA levels should be undetectable within four weeks, and early testing at 4–8 weeks after surgery could be useful for determining patient prognosis [6]. After 4–8 weeks following RP, 5–20% of PC patients show detectable or persistent PSA > 0.1 ng/mL [7]. It may result from persistent local disease, pre-existing metastases, or residual benign prostate tissue.

Different definitions of PSA kinetics (PSA-DT and PSA velocity) have been used to stratify patients with BCR into low- or high-risk for early clinical or metastatic progression [8]. In non-metastatic castration resistant PC (nmCRPC), PSA-DT has been associated with metastasis-free survival and overall survival (OS) results and is used to identify high-risk nmCRPC patients who benefit from intensified therapy (PSA-DT threshold < 10 months) [9].

According to the results of a systematic review and meta-analysis conducted by Van den Broeck et al., patients with BCR after primary treatments have a higher risk of developing distant metastases and prostatic cancer-specific and overall mortality (OM) [10]. Nevertheless, the effect size of BCR as a risk factor for mortality is extremely variable [11]; studies report an increased risk of mortality ranging from HR 1.03 (95% CI: 1.004–1.06) to HR 2.32 (95% CI: 1.45–3.71) [12], whereas after primary RT, the increased risk of prostate cancer-specific mortality (PCSM) ranges from HR 1.34 to HR 1.45 [13]. In this systematic review, different clinical and pathologic variables (e.g., T-category, PSA, ISUP grade) and PSA kinetics (PSA-DT and interval to PSA failure) have been used to significantly predict the probability of future metastases, PC-specific mortality, and OM [10]. Recently, Falagario et al., using 16,311 PC cases, evaluated the association of BCR after RP or RT with PCSM [13]. Following RP, the 15-year cumulative incidences of BCR were 16% (95% CI, 15–18%) in low-risk PC, 30% (95% CI, 27–32%) in the intermediate-risk group, and 46% (95% CI, 42–51%) in high-risk PC. Following RT, the 15-year cumulative incidences of BCR were 18% (95% CI, 15–21%) in low-risk PC, 24% (95% CI, 21–26%) in the intermediate-risk group, and 36% (95% CI, 33–39%) in high-risk PC. The 10-year cumulative incidences of prostate cancer-specific mortality (PCSM) after RP and RT were 4% (95% CI, 2–6%) and 24% (95% CI, 19–29%) in low-risk BCR (Gleason score < 8 and PSA-DT > 12 mo) and 9% (95% CI, 5–13%) and 46% (95% CI, 40–51%) in high-risk BCR (Gleason score ≥ 8 or PSA-DT ≤ 12 mo), respectively. In patients with BCR after RP, the following outcomes were found to be significantly correlated with PCSM and OM: high pathologic ISUP grade, short interval to biochemical failure, short PSA-DT, positive surgical margins, and high pT category. The corresponding results for patients with BCR following RT as predictors of PCSM and OS were short interval to biochemical failure; distant metastatic recurrence; high biopsy ISUP grade; high cT category; short interval to biochemical failure; and high initial (pre-treatment) PSA.

In the future, genetic biomarkers such as the Decipher genomic classifier (GC) might be routinely used as adjuvant indicators of high-risk BCR. Dal Pra et al. evaluated samples from 226 RP patients to determine the association of the GC with the 5-year freedom from biochemical progression (FFBP) [14]. Patients with GC high had a 5-year FFBP of 45% (95% CI 32–59%) versus 71% (95% CI 64–78%) in GC low–intermediate.

Homologous recombinant repair (HRR) pathway of the DNA damage repair (DDR) gene family and in particular BRCA1-2 pathogenetic variants (PV) are now used to tailor therapy with poly-ADP-ribose polymerase (PARP) inhibitors in metastatic CRPC. An early determination of somatic HRR PVs in non-metastatic PC could have a prognostic impact on the oncological outcomes of these patients and could help to better define high- and low-risk cases for metastatic clinical progression. Castro et al. evaluated the prognostic value of BRCA PV in terms of risk of metastatic progression and cancer-specific survival after RP or RT in localized PC [15]. At 3-, 5-, and 10-year intervals following primary treatments, 97%, 94%, and 84%, respectively, of BRCA- and 90%, 72%, and 50%, respectively, of BRCA+ cases were free of metastasis (*p* < 0.001). The cancer-specific survival rates were significantly better in the BRCA- cohort (99%, 97%, and 85%, respectively, at 3, 5, and 10 years) than in the BRCA+ cohort (96%, 76%, and 61%, respectively; *p* < 0.001). Also, according to multivariate analysis, the risk of metastasis-free survival (MFS) was significantly lower in BRCA+ cases (HR 2.36; CI 1.38–4.03; *p* = 0.002).

## 3. Imaging for Biochemical Recurrence

In the event of BCR, the key question that remains is whether the PSA rise reflective of metastatic disease or a consequence of a locally confined recurrence. With the development of new metastasis-directed therapies (MDTs) and the possibility of local treatments even for metastatic patients with a low-disease burden, correct identification and the use of imaging is essential for tailored treatment planning [16].

A recent systematic review by De Visschere et al. evaluated the role of the existing imaging techniques in early recurrent PC [17]. A total of 98 studies evaluating transrectal ultrasonography (TRUS), computed tomography (CT), bone scintigraphy (BS), multiparametric magnetic resonance imaging (mpMRI), whole-body MRI (wbMRI), and positron emission tomography (PET)-CT/MRI using different tracers were included. TRUS is rarely used in this setting and the few studies evaluating its efficacy present conflicting results. Despite that, color/power Doppler, contrast-enhanced TRUS, and multiparametric ultrasound are emerging as adjuvant techniques to increase the sensitivity and specificity of grayscale TRUS [18]. The authors found three studies reporting the detection rates of CT in the setting of early PC recurrence. Sensitivity and specificity were very low for both local recurrence and distant metastasis detection, meaning that morphological and size criteria are not enough, especially for lymph nodes or for low metastatic burden. Despite the non-unanimous results presented by De Visschere et al., mpMRI shows good accuracy when it comes to local recurrence detection, even with low PSA levels. With PSA levels ≤ 5.0 ng/mL, positivity rates ranged from 66.7% to 86.7% and sensitivity was around 90%, but the detection rate was good even in subgroups with a lower PSA threshold. Panebianco et al. obtained a sensitivity of 94–100% and a specificity of 92–97% in 116 patients with recurrent PSA values of 1.4–2.9 ng/mL; while in a study comparing mpMRI with 18F choline PET-CT for local recurrence, the former showed a sensitivity of 92.0%, a specificity of 75.0%, and a positivity rate of 82.1% with PSA levels of 0.8–1.4 ng/mL [19,20]. BS has been widely used for the detection of bone metastases in PC patients. De Visschere et al. found three studies reporting detection rates of BS in the early recurrence setting. The detection rate ranged from 10% in cases with a recurrent PSA value of <1.0 ng/mL to 20% when a level of <4.0 ng/mL was considered [17]. 18F FDG-PET-CT has not been widely used in the setting of PC, mainly because of the low glucose metabolism of PC cells and the accumulation of the tracer in the urinary system [21]. A 2020 retrospective analysis by Michaud et al. evaluated the role of 11C-choline PET/CT in detecting BCR of PC in 282 cases [22], scoring the level of suspicion as 0, negative; 1, equivocal; and 2, positive by two readers. A score of 2 was found in 28%, 46%, 62%, and 81%, respectively, in patients with PSA levels of <0.5 ng/mL, 0.5–0.99 ng/mL, 1.0–1.99 ng/mL, and ≥2.0 ng/mL. 18F (fluoro)choline is another widely studied PET tracer for PC. For local recurrence, De Visschere et al. found detection rates ranging from 16.7% to 76.0% in patients with BCR after primary treatment and a PSA level < 1 ng/mL, which are lower than that demonstrated by mpMRI [17]. Regarding bone metastasis, 18F choline PET-CT appears to have better detection rates when compared with BS, mainly because of early bone infiltration detection, before the occurrence of osteoblastic reactions. To date, the most promising data are those generated with radiotracers targeting the PSMA, which is overexpressed in most PC cells. A 2022 systematic review by Mazrani et al. included 20 prospective studies evaluating the role of 68Ga and 18F PSMA PET/CT and PET/MRI in 2110 PC cases with BCR. Pooled PSMA PET positivity was 66.6% and the only factor significantly associated with PSMA PET positivity was PSA level at the time of the analysis [23].

After RP, 68Ga PSMA-11 PET/CT demonstrates good detection rates even at very low PSA levels. De Visschere et al. reported positivity rates ranging from 11.0% to 65.0% with a PSA serum level < 0.5 ng/mL [17]. The largest available series was published by Afshar-Oromieh et al. reporting a 43.3% detection rate in 226 patients with PSA ≤ 0.2 ng/mL after RP [24]. In the setting of BCR after primary RT, 68Ga PSMA PET/CT accuracy differs according to the PSA level at the time of the exam. Einspieler et al. reported a 90.7% detection rate for 68Ga PSMA PET/CT in 118 patients with BCR after primary RT and a median PSA of 6.4 ng/mL; the detection rates were, respectively, 81.8%, 95.3%, and 96.8% for PSA values of 2 to <5, 5 to <10, and ≥10 ng/mL [25]. The detection rate of 68Ga PSMA PET/CT can vary depending on the metastatic site. In a systematic review by Eissa et al., detection rates reached 74.5%, 89.7%, 61%, and 14.3% for prostate recurrence, lymph nodes, bone metastasis, and visceral metastasis, respectively [26]. Specifically for lymph node recurrence, conventional imaging has limited value with a pool sensitivity and specificity of 42% and 82% for CT and 39% and 82% for MRI [27]. Studies by Rauscher et al. and Jilg et al. reported the detection of lymph node recurrence using 68Ga PSMA PET/CT with positive predictive values of 94.6% and 99.1% and negative predictive values of 87.8% and 92.3%, respectively [28,29].

In summary, the ability of the currently available imaging modalities in the detection of both local and metastatic recurrence in the setting of BCR after primary treatments is still highly dependent on PSA levels. The strength of the recommendation of the current EAU guidelines in this setting is still weak. In the event of PSA-only recurrence after RP, PSMA PET/CT seems to be the imaging modality with the highest sensitivity at low PSA levels (<0.5 ng/mL) [30]. Results of PSMA PET/CT may help distinguish patients with local recurrences in the prostatic fossa from those with low-volume distant metastases. After RT, the high sensitivity of mpMRI may help in the detection of local recurrences, in case of a local salvage treatment indication [31]. Nonetheless, systemic staging is still needed before treatment initiation in the event of local recurrence, because of the high morbidity of post-RT salvage therapies. Choline-, fluciclovine-, or PSMA-PET/CT can all be used in these patients, but again, the highest sensitivity of PSMA PET/CT is preferred, in order to more accurately rule-out any distant metastasis.

## 4. How Biochemical Progression Is Treated Today

### 4.1. BCR after RP

There is still limited evidence and controversy regarding the correct timing and treatment in patients with PSA-only recurrence after RP (Table 2). Salvage radiotherapy and ADT are the treatment modalities accepted by current guidelines, while other options, such as salvage pelvic lymph node dissection (sPLND) in the event of nodal recurrence, are still awaiting validation [32].

Salvage RT (SRT) has been shown to decrease the onset of distant metastasis and improve PC-specific mortality, providing a possibility of cure in patients with increased PSA after RP. In post-RP patients with PSA levels over 0.1–0.2 ng/mL, the RAVES and RADICAL trials demonstrated 5-year BCR-free survival rates of 88% for SRT [33,34]. A ten-year-older study by Boorjian et al. evaluating the impact of SRT on disease progression and survival showed that SRT decreased the risk of local recurrence (HR 0.13, 95% CI 0.06–0.28, *p* < 0.0001) and delayed hormonal therapy (HR 0.81, 95% CI 0.71–0.93, *p* = 0.003) and systemic progression (HR 0.24, 95% CI 0.13–0.45, *p* < 0.0001), when compared to non-SRT [35]. Pre-SRT PSA has been proven to be one of the main prognostic factors for treatment response. In a 2012 study by Siegmann et al., patients with a PSA level of <0.28 ng/mL before SRT had a higher two-year failure-free survival and no evidence of disease (bNED) than those with a pre-SRT PSA level of >0.28 ng/mL (78% vs. 61% respectively) [36]. Two systematic reviews confirmed the role of PSA level in predicting the 5-year BCR-free survival after SRT [37,38]. In the analyzed studies, the probability of achieving an undetectable PSA level was >60% when treatment was commenced with a PSA < 0.5 ng/dl. In addition to PSA, the ISUP grade, margin status, and pT stage seem to have an impact on metastasis-free and overall survival [39].

Based on the current guidelines, it is still uncertain whether the addition of ADT to SRT in patients with PSA-only recurrence can improve OS and progression-free survival (PFS), and treatment modalities and duration are not yet standardized. Molecular imaging advancements are now revealing that greater pre-SRT PSA levels are associated with an increased risk of extra-pelvic metastatic illness. In this scenario, it is reasonable to believe that patients with a higher pre-SRT PSA level may have an increased benefit from ADT for occult metastasis. Data from the RTOG 9601 suggest a cancer-specific survival (CSS) and OS benefit with the addition of 2 years of Bicalutamide to early SRT [40]. In a secondary analysis of this multicenter, double-blinded, placebo-controlled, randomized clinical trial, ADT was associated with an OS benefit in patients with a PSA level greater than 1.5 ng/mL (25% 12-year absolute benefit; HR 0.45; 95% CI 0.25–0.81), but not in those with a PSA level of 1.5 ng/mL or less. Moreover, in patients receiving early SRT (PSA < 0.6 ng/mL), there was no improvement in OS but an increased OM risk (HR 1.94; 95% CI, 1.17–3.20) and a higher incidence of late grade 3 to 5 cardiac and neurologic side effects, when ADT was added [41]. A systematic review of the benefit of combining ADT with SRT proposed a risk stratification of patients, in order to individualize treatment, based on pre-SRT PSA level (0.5, 0.6–1, >1 ng/mL), margin status, and ISUP grade [42]. Regarding the duration of ADT, data from a recent multicenter study suggested a significant effect of long-term ADT in patients with two or more adverse features (pT stage ≥ pT3b, pathologic Gleason ≥ 8, and PSA level at SRT > 0.5 ng/mL). Short-term ADT, on the other hand, was sufficient in patients with a single risk factor, whilst those without any risk factors did not benefit significantly from simultaneous ADT [43].

The oncological role and safety of sPLND in the era of modern imaging was evaluated by a recent systematic review by Ploussard et al. The reference imaging technique was PSMA or choline PET/CT and mean follow-up was for 29.4 months. The 2- and 5-year biochemical PFS rates ranged from 23% to 64% and from 6% to 31%, respectively, and 5-year OS was 84% [44]. However, despite the interest in the role and safety of sPLND in nodal recurrence, high-level evidence is still lacking.

### 4.2. BCR after RT

Treatment options for BCR after RT include re-irradiation, local procedures, and a ‘wait and see approach’. The choice should be based on available strategies and the EAU risk groups. Salvage re-irradiation options include stereotactic ablative body radiotherapy (CyberKnife^®^ US, or linac-based treatment) and salvage brachytherapy [either High Dose Rate (HDR) or Low Dose Rate (LDR)]. Stereotactic ablative body radiotherapy (SBRT) should be offered to patients with a good IPSS score and a biopsy proving local recurrence. For the linac-based approach, a prospective single-center study by Bergamin et al. showed a 2-year BCR-free rate of 80% with a total dose (TD) of 36–38 Gy delivered in fractions of 6–6.2 Gy [45]. For the Cyberknife treatment, two large retrospective series of 50 and 100 patients with a TD of 34 and 36 Gy demonstrated, respectively, a 5-year BCR-free survival rate of 60% and a 3-year BCR-free survival rate of 55% [46,47]. In a systematic review and meta-analysis of local salvage therapies after radiotherapy for prostate cancer (MASTER), SBRT showed severe genitourinary (GU) toxicity and gastrointestinal (GI) toxicity rates of 4.2% and 0.0%, respectively, which are lower than that of any other local salvage approach [48]. Regarding brachytherapy, in the MASTER trial, the 2-year and 5-year BCR-free survival rates for HDR and LDR were, respectively, 77% and 60% and 81% and 56%. With rates of severe GU toxicity of 8% for HDR and 8.1% for LDR and rates of severe GI toxicity of 0% for HDR and 1.5% for LDR, brachytherapy proved to be safer than salvage RP or salvage HIFU, but not safer than salvage SBRT.

Compared to primary surgery, salvage RP after RT is associated with a higher risk of anastomotic stricture (47% vs. 5.8%), urinary retention (25.3% vs. 3.5%), and rectal injury (9.2 vs. 0.6%) as well as a higher probability of poor long-term functional outcomes such as urinary incontinence and erectile dysfunction [49]. In a recent multicenter retrospective study of 414 salvage RP patients with a median follow-up period of 36 (IQR 20.4–60.5) months, 59.8% (*n* = 229) of men did not experience BCR, 9.4% (*n* = 39) had disease persistence after salvage RP, and 30.9% (*n* = 115) had BCR (median time of BCR being 12 (IQR 5.75–30) months from surgery) [50]. In an older systematic review, Chade et al. demonstrated a similar BCR-free survival (47–82%) and a 10-year BCR-free survival and OS of 28–53% and 54–89%, respectively [51]. Currently, no data support the use of salvage RP in patients with a negative biopsy. Ideal candidates for this salvage surgical treatment are patients with a positive pre-treatment biopsy, T1–T2 stage before RT, no evidence of distant metastasis or lymph-node involvement, PSA < 10 ng/mL, and a life expectancy >10 years.

Alternatives to SRT and salvage RP are salvage cryoablation of the prostate (SCAP) and salvage high-intensity focused ultrasound (S-HIFU). A retrospective study comprising 418 patients with BCR and local recurrent disease after EBRT treated with S-HIFU showed a 5-year BCR-free survival of 49% [52]. Two similar studies with shorter follow-up periods demonstrated comparable results in terms of BCR-free rate [53,54]. Urine incontinence, urine retention, rectourethral fistula, and erective dysfunction are the most common side effects associated with S-HIFU, and the MASTER trial adjusted pooled analysis for severe GU toxicity was 22.66% (95% CI: 16.98–28.85%) [48]. As state-of-the-art treatment, due to the lack of high-certainty data, one should only offer S-HIFU in a clinical trial setting. Regarding SCAP, one of the largest series currently available included 898 patients with BCR after primary RT. The 5-year BCR-free probability was 71.3% [55]. Less optimistic results were proposed by the MASTER systematic review, with an adjusted pooled analysis for 5-year BCR-free survival of 50.25% in a total of 32 studies and 5513 patients [48].

### 4.3. Active Observation

Despite the indication for salvage treatments, a ‘wait and see‘ strategy remains an option for the EAU BCR ‘Low-Risk’ group. For instance, studies report that only approximately 30% of patients with BCR after primary surgery will eventually develop clinical recurrence, with only 16.4% dying from PC [10]. An observational study of 407 patients experiencing BCR following RP used PSA kinetics and DT pattern to evaluate the need for adjuvant treatment (ADT or SRT) or active observation (AO) with close monitoring of PSA and DT. Risk assessment was performed using PSA-DT (>12 vs. <12 months), and PC cases with rapidly decreasing DTs were assigned to treatment [56]. The PCSM in the treatment group (TG) was 10.7%, whereas in the AO group, it was 0% (*p* < 0.001). An initial PSA-DT > 12 months was present in 73.6% of the AO group versus 22.6% of the TG group (*p* < 0.001). In the AO group, an increasing PSA-DT trend was seen in 71.5%, whereas in the treatment group, it was in only 32.7% of the cases (*p* < 0.001). Using this selection, 33% of patients with BCR were managed conservatively without compromising their life expectancy. The key benefit of AO is avoiding overtreatment, with a positive impact on the quality of life and reduced side effects, in addition to the monetary savings by avoiding ADT and RT and their side effects’ management.

### 4.4. Systemic Treatment

Conflicting results were published regarding the clinical effectiveness of early ADT in the setting of BCR after curative therapy of primary PC without overt clinical disease. The TOAD trial is a multicenter, phase 3 trial including 261 patients with PSA relapse after primary treatment (group 1) and 32 with non-curable disease (group 2) randomized on a 1:1 ratio to delayed ADT (delayed-therapy arm) or immediate ADT (immediate-therapy arm) [57]. The median follow-up period was 5 years. Specifically for group 1, 26 men (19%) assigned to the delayed-therapy arm and 14 men (11%) assigned to the immediate-therapy arm died. The estimated 5-year OS rates were 78.2% (95% CI 67.2–85.8) in the delayed-therapy arm and 84.3% (73.9–90.8) in the immediate-therapy arm. The TOAD study was the only randomized clinical trial (RCT) to report a favorable but limited effect of early ADT in a subsequent systematic review on this topic, while the majority of the other studies reported no differences in terms of OS between early and delayed ADT. No data were found on the effectiveness of different types of ADT [58]. Due to the lack of clear effectiveness and the considerable adverse effects, early ADT should only be considered in patients with the highest risk of disease progression (short PSA-DT and high initial ISUP grade) and a life expectancy >10 years.

Intermittent androgen deprivation (IAD) for PSA elevation after primary treatment (RP or RT) was proposed to improve the quality of life and delay hormone resistance, without negatively impacting life expectancy. A 2012 RCT by Crook et al. randomly assigned 1386 patients with BCR in a 1:1 ratio to either intermittent or continuous therapy [59]. The median follow-up period was 6.9 years. The median OS was 8.8 years and 9.1 years in the IAD group and in the continuous-therapy group, respectively. The hazard ratio (HR) for death with IAD versus continuous therapy was 1.02 (95% CI, 0.86 to 1.21). At the baseline, there were no meaningful differences in the quality-of-life scores between the two groups. At 5 years, IAD was associated with significantly better scores for hot flashes (*p* < 0.001), desire for sexual activity (*p* < 0.001), and urinary symptoms (*p* = 0.006). An important limitation of this RCT is the lack of any stratifying criteria such as PSA-DT or initial risk factors. In another RCT of 77 patients, there was no difference in terms of disease progression and quality of life between IAD and CAD after a median follow-up period of 48 months [60].

## 5. How Systemic Treatment for BCR Can Condition the Development of a Non-Metastatic CRPC

An indiscriminate use of ADT in patients with only BCR can produce an aberration of the natural history of PC, with the development of castration resistance (CR) that precedes that of metastases. CRPC is defined as the association of castrate serum testosterone level < 50 ng/dL or 1.7 nmol/L and either biochemical progression (three consecutive rises in PSA levels, resulting in two 50% increases over the nadir, and a PSA level > 2 ng/mL) or radiological progression (>two bone metastases identified by bone scan or a visceral metastasis defined by the RECIST criteria) [61,62]. The annual incidence of nmCRPC in the United States has been estimated at roughly 60,000 cases in 2020, with a 16% annual OM [63]. Prior to 2018, no treatment approach for non-metastatic patients with a PSA rise during ADT (nmCRPC) had demonstrated an OS benefit [64].

For asymptomatic nmCRPC patients, the Prostate Cancer Radiographic Assessments for Detection of Advanced Recurrence (RADAR) group guidelines recommend PSA testing every 3 months and evaluation by conventional imaging at PSA levels of 2 ng/mL and 5 ng/mL and then every time the PSA level doubles [65]. In a 2019 study, PSMA-PET detection rate for pelvic disease and distant metastasis was evaluated on 200 high-risk nmCRPC patients. PSMA-PET detected any disease in nearly all patients (196 of 200) and M1 disease in 55% of patients previously diagnosed with nmCRPC [66]. Although PSMA-PET is widely used in clinical practice also for initial staging or for BCR, current guidelines do not endorse its indication beyond these settings. It is important to note that, despite the high probability of micrometastatic disease in high-risk nmCRPC defined by conventional imaging, this did not preclude a survival benefit in the three studies evaluating novel treatment options for nmCRPC defined by the conventional imaging [67,68,69].

Few studies have analyzed risk factors for the development of CR in BCR non-metastatic patients. A recent study by Salciccia et al. retrospectively evaluated, in 170 PC cases with BCR after primary treatments, the impact of continuous ADT (CAD) and intermittent ADT (IAD) on the development of CRPC in both metastatic and non-metastatic settings (PET/CT was used as imaging). According to univariate analysis, the use of continuous administration of ADT significantly increases the HR for CRPC-M0 progression (HR 3.48; 95% CI 1.66–7.29; *p* = 0.01) when compared to IAD administration, either in cases treated with RP or RT, and the result remains significant also with multivariate analysis (RR 2.34, 95% CI 1.52–5.33; *p* = 0.03) [70].

Similar to the BCR PC group, nmCRPC is a heterogeneous condition, ranging from indolent disease to aggressive forms that rapidly progress to symptomatic metastases, with significant differences in terms of OS and CSS [71]. In a study on 201 patients with nmCRPC, at 2 years of follow up, only 33% of patients developed radiologically evident metastases (on conventional imaging), and the median bone metastasis-free survival (BMFS) was 30 months. A baseline PSA level greater than 10 ng/mL and PSA velocity independently predicted shorter time to first bone metastasis, OS, and metastasis-free survival [9]. A more recent phase 3 randomized trial of denosumab in nmCRPC suggested that a PSA-DT of ≤10 months predicted a shorter OS and BMFS [72]. Of the above-mentioned parameters, PSA-DT is considered the most relevant predictive factor of disease progression, supporting its use in the selection of high-risk patients [73].

Delaying the onset of metastasis and thus increasing OS are the main treatment goals for nmCRPC. Prior to 2019, the NCCN guidelines recommended observation for patients with PSA-DT > 10 months, given the low risk of progression, and for those with PSA-DT ≤ 10 months, it recommended enrolling them in a clinical trial [74]. The first two new-generation androgen receptor signaling inhibitors (ARSIs) to be approved for nmCRPC were apalutamide and enzalutamide in 2018. In the following year, FDA and EMA also approved the use of Darolutamide in this setting. The PROSPER, SPARTAN, and ARAMIS trials are all phase 3, randomized, double-blinded, placebo-controlled studies on high-risk nmCRPC (defined as PSA-DT ≤ 10 months and PSA level ≥ 2 ng/mL) using metastasis-free survival (MFS) as the primary endpoint [67,68,69]. The definition of non-metastatic disease was obtained using conventional imaging and all the three ARSIs demonstrated a significant MFS benefit when compared to the ADT arm. No direct comparison of the three aforementioned agents has been published; indirect comparisons from three meta-analyses suggest a similar effectiveness, whereas darolutamide seems to have better tolerability [75,76,77].

## 6. Differences between Real World and Clinical Trials in the Management of Biochemical Recurrence

Real-world data (RWD) are data gathered outside of RCTs that reflect actual clinical practice. While RCTs provide the highest degree of evidence for demonstrating the efficacy of medical therapies, studies using RWD provide further data concerning the effectiveness, long-term results, and safety of those interventions in real-world situations [78]. The significance of RWD in in-depth investigation of PC etiology, diagnosis, and treatment efficacy is expanding as a result of the establishment of high-quality clinical national and worldwide registers. However, real-world studies to verify the results obtained in RCTs continue to remain little used, particularly in the setting of BCR PC. A recent 2021 review demonstrated the value of a high-quality national register, the National Prostate Cancer Register (NPCR) of Sweden, in generating high-quality evidence in different PC settings, such as etiology, treatment, and adverse events’ management [79].

After RP, the literature describes a >30% risk of developing BCR. A recent American retrospective cohort study used RWD extrapolated from nationally representative Optum© Electronic Medical Records (EMR) from 2010 to 2021 [80]. The study included a total of 15,198 patients, and the median follow-up period was 3.9 years. RT after primary treatment was initiated in 14.3% of patients, either adjuvant or salvage. BCR occurred in 19.8% of patients, and among patients with BCR, 6.3% had a PSA-DT ≤ 10 months, 17.1% developed metastasis, 13.8% developed CRPC, and 9.7% died. With multivariate analysis, authors found that a PSA level ≥ 20 ng/mL, Charlson Comorbidity Index (CCI) > 0, age ≥ 65, and the African-American race were the most relevant baseline risk factors for BCR.

Regarding the staging of patients with a PSA rise after primary treatment, a 2023 study by Burgard et al. evaluated the real-world efficacy of PSMA-PET in detecting tumor localization after early (PSA level ≤ 0.2 ng/mL) BCR [81]. Authors conducted a retrospective analysis in 115 men with BCR after RP for intermediate- or high-risk PCs at very a low PSA level (≤0.2 ng/mL) and a PSA-DT ≤ 12 months. The primary endpoint was the per-patient visual detection rate (positive scans/total scans). Secondary endpoints included the detection rate by lesion type, PSA concentration, PSA-DT, and Gleason score. A total of 25.2% of patients had lesions suspicious for PC recurrence with PSMA-PET/CT with a total of 44 lesions, including 11 putative local recurrences, 22 putative lymph node metastases, and 11 putative bone metastases. When comparing these data with the available literature, the detection rate seems to be significantly lower than that demonstrated by the largest available series by Afshar-Oromieh et al. (43.3% detection rate in 226 RP patients with PSA ≤ 0.2 ng/mL) [24]. In contrast, these RWD results are in line with those demonstrated in a 2020 meta-analysis (33% detection rate in 197 RP patients) [82] and even better than those reported by Meredith et al. in 2016 [83].

In 2021, data from a nationwide US survey on treatment modalities for nmPC were published. The physicians included were board-certified urologists and oncologists with at least five years of clinical practice experience and having treated at least 30 PC patients monthly [84]. At final analysis, 4415 patients with nmPC were stratified by stage. After 5 years following the initial therapy, relapse occurred in 22.1%, 31.4%, 43.6%, and 60.4% in stage I, II, III and IV (M0) patients, respectively. Stage I and II patients were most likely to experience biochemical progression only, while metastatic recurrence was considerably more frequent among stage IV (M0) patients (41.2%). Regardless of the initial treatment, more than half of the recurrent patients received systemic therapy with or without RT; only roughly 10% of patients with recurrence were addressed to AS, and following a relapse after RP, 49% and 17.6% of patients underwent only systemic therapy or systemic therapy + RT, respectively.

In 2023, Tilki et al. used multivariable Cox regression analysis on a multinational database of 25,551 patients with pT2-4N0M0 or NXM0 PC after RP to evaluate the existence of a prespecified PSA level associated with an increased all-cause mortality (ACM) at the beginning of SRT [85]. ACM was evaluated at a starting PSA of 0.10 ng/mL and then at every 0.05 ng/mL increase up to 0.5 ng/mL. A total of 1556 (6.09%) cases were submitted to SRT at a low PSA level ≤ 0.25 ng/mL, whereas 1677 (6.56%) were submitted when the PSA level was > 0.25 ng/mL. In total, 39.65% of cases with a PSA level > 0.25 ng/mL was submitted to ADT as compared to 27.44% cases with a PSA level ≤ 0.25 ng/mL. At a median follow-up of 6 years, patients submitted to SRT at a PSA level > 0.25 ng/mL had a significantly higher ACM risk (AHR, 1.49; 95% CI, 1.11 to 2.00; *p* = 0.008) compared with those submitted to SRT at a PSA level ≤ 0.25 mg/mL.

Tilki et al. showed no significant differences in ACM risk between adjuvant RT and salvage SRT at PSA level ≤ 0.25 ng/mL in patients with one high-risk factor (Gleason score 8–10 or pT3/4). Similarly, the Radiotherapy and Androgen Deprivation after Local Surgery-RT randomized trial demonstrated no superiority of adjuvant to early (PSA < 0.25 ng/mL) salvage post-RP SRT in terms of disease-free survival [34].

## 7. The Concept of Anticipation and Incrementation in the Treatment of Biochemical Recurrence

**Figure 1:**
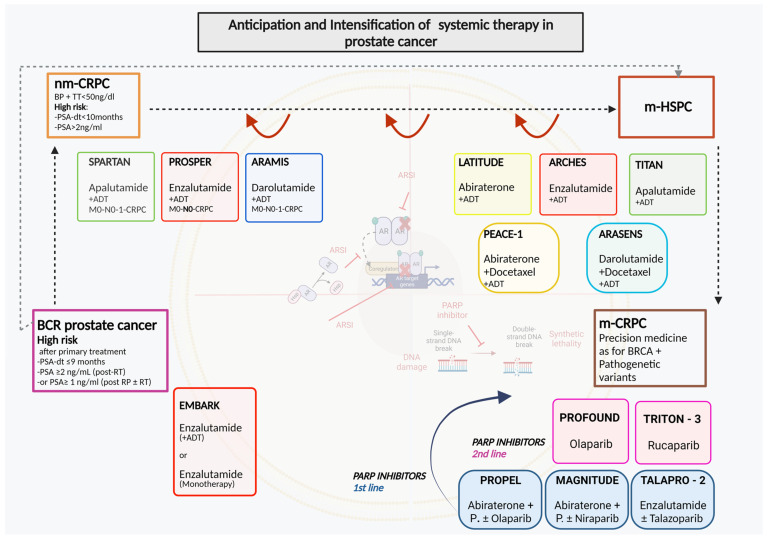
Anticipation and intensification of systemic therapy in prostate cancer [86,87,88,89,90,91,92,93,94,95]. ADT: androgen deprivation therapy; AR: androgen receptor; ARSI: androgen receptor signal inhibitor; BCR: biochemical recurrence; HSP: heat shock protein; m-CRPC: metastatic castration-resistant prostate cancer; m-HSPC: metastatic hormone-sensitive prostate cancer; nm-CRPC: non-metastatic castration-resistant prostate cancer; P.: prednisone or prednisolone; PARP: poly (ADP-ribose) polymerase.

ARSI therapies in PC have led to indications increasingly aimed at a concept of anticipation and intensification of therapy starting from the CRPC phase and then completely involving the metastatic HSPC phase. Recently, this concept has been proposed in even earlier phases of the disease as neoadjuvant to primary treatments and in case of BCR after primary therapies (Figure 1).

### 7.1. EMBARK Study

The EMBARK study is probably one of the most innovative studies to date analyzing the anticipated role of ARSIs in PC patients with BCR after primary treatment [96]. The study uses the concept of PSA-DT to select only non-metastatic PC with high-risk BCR.

The EMBARK is a randomized, phase III study focused on nmHSPC patients with a PSA-DT of ≤9 months, screening PSA level of ≥1 ng/mL post RP (+/−RT) or ≥2 ng/mL above nadir post-RT, and serum testosterone level ≥ 150 ng/dL. The PSA value used to start therapy after RT corresponds to that defined as BCR; on the contrary, in patients undergoing RP, it expects a value higher than the normally used cutoff of 0.2 ng/mL. Men are randomized 1:1:1 into enzalutamide 160 mg/d plus leuprolide (LHRH analogue) or placebo plus leuprolide in double-blind arms or enzalutamide monotherapy (open label). The EMBARK represents the first study where a treatment with an ARSI that replaces and is not only associated with ADT is proposed. In this direction, it has the courage to analyze whether the inclusion of ARSIs in such an early phase of the disease such as BCR can determine the unsuitability of the classic ADT. Patients have treatment suspended on week 37 if PSA levels are <0.2 ng/mL and reinstated if levels increase to ≥2.0 ng/mL (RP) or ≥5.0 ng/mL (RT). Patients with a PSA level ≥ 0.2 ng/mL stay on the treatment until the requirements to stop it are satisfied. This interruption of therapy in the event of a reduction in PSA levels to undetectable values suggests the use of a non-continuous treatment but does not represent a true intermittent strategy like IAD. The primary endpoint is metastasis-free survival (MFS) comparing enzalutamide plus LHRHa vs. placebo plus LHRHa. Key secondary endpoints are MFS in the enzalutamide monotherapy group in comparison with the other two arms, time to PSA progression, time to first use of new antineoplastic therapy, and overall survival. Progression-free survival on the first subsequent therapy (PFS2) is an exploratory endpoint. The study confirms that the maintenance of a non-metastatic disease stage must be considered the primary endpoint in this disease phase with only BCR, similarly to what is considered in trials on patients with nmCRPC. A total of 1068 randomized patients were followed-up with for a median of 60.7 months. The 5-year MFS was 87.3% (95% confidence interval [CI], 83.0 to 90.6) in the combination group, 71.4% (95% CI, 65.7 to 76.3) in the leuprolide-alone group, and 80.0% (95% CI, 75.0 to 84.1) in the enzalutamide monotherapy group. Either the combination enzalutamide plus leuprolide or enazalutamide monotherapy was superior to leuprolide alone (HR 0.42; 95% CI, 0.30 to 0.61; *p* < 0.001 and HR 0.63; 95% CI, 0.46 to 0.87; *p* = 0.005, respectively). Compared to leuprolide alone, patients treated with enzalutamide plus leuprolide demonstrated a significant reduction in the risk of PSA progression, longer time to the first use of new antineoplastic therapy, and above all a longer OS (HR 0.59; CI 038–0.91; *p* < 0.02). Compared with leuprolide alone, patients treated with enzalutamide alone demonstrated a significant reduction in the risk of PSA progression and longer time to the first use of new antineoplastic therapy, but no significant advantage in terms of OS (HR 0.78; CI 0.52–1.17; *p* = 0.23). These results were maintained regardless of the type of primary treatment used (RP or RT).

### 7.2. Ongoing Trials

Aggarwal et al. during the 2022 ESMO annual meeting presented the PRESTO trial, a randomized phase III, open-label trial in patients with BCR PC and PSA-DT ≤ 9 months, M0 at conventional imaging (no PET-CT imaging was used) [97]. The primary endpoint was BCR-free survival and secondary endpoints included safety, quality of life, MFS, and castration resistance. Patients (stratified in PSA-DT < 3 vs. 3–9 months) were randomly treated with a 52-week course of ADT, ADT + apalutamide, or ADT + apalutamide + abiraterone acetate plus prednisone. The enrolment started in March 2017, including a period in March 2020 where the COVID-19 pandemic led to a mitigation plan with flexible LHRH analog dosing schedules. A total of 504 patients were randomly assigned to ADT alone (*n* = 167), ADT + apalutamide (*n* = 168), or ADT + apalutamide + abiraterone acetate plus prednisone (*n* = 169). At a median follow-up of 21.5 months, both combination arms significantly prolonged BCR-free survival when compared to the ADT alone group. In particular, the median BCR-free survival was 24.9 months for ADT + apalutamide vs. 20.3 months for ADT (HR 0.52 (95% CI 0.35–0.77)) and 26.0 months for ADT + apalutamide + abiraterone acetate plus prednisone vs. 20.0 months for ADT (HR 0.48 (95% CI 0.32–0.71)). Hypertension was the most common grade ≥ 2 adverse event (19.4%, 23.4%, 30.4% in ADT, ADT + apalutamide, and ADT + apalutamide + abiraterone acetate plus prednisone arms, respectively). Treatment was concluded because of adverse events in only 1.8% cases across all treatment arms.

The ARASTEP is an ongoing, randomized, double-blind, phase 3 study evaluating the use of darolutamide compared to placebo in patients with high-risk BCR PC. Patients (estimated enrollment, *n* = 750) will be randomized to darolutamide 600 mg twice daily (BID) or placebo BID, both in combination with ADT, for a maximum duration of 24 months or until disease progression and severe toxicity [98].

The ARAMON is an ongoing, open-label, phase 2 study evaluating the use of darolutamide and enzalutamide as monotherapy in the treatment of patients with PC experiencing BCR following definitive treatment for localized disease [99]. During the lead-in phase of the study, 25 patients will receive darolutamide 600 mg BID. A testosterone assessment will be conducted at 12 weeks. The randomized phase of the study will only proceed if, during the lead-in phase, the testosterone level criteria will be respected. The randomized phase will enroll approximately 40 patients randomized 1:1 to darolutamide 600 mg BID or enzalutamide 160 mg once daily. In either phase, patients will receive treatment for 52 weeks or until unacceptable toxicity, PSA progression, or death.

## 8. Conclusions

The BCR phase is extremely heterogeneous and at least a stratification into low- and high-risk cases for early progression in metastatic disease is necessary. PSA-DT currently represents the best parameter to define low- and high-risk BCR PC; however, other factors (T stage, ISUP grading, and genetic pattern) can be considered through risk calculators or nomograms. After the identification of biochemical relapse, new generation imaging, especially PSMA PET/CT, is needed to exclude the presence of low-volume metastasis, because of the different therapeutic approaches currently indicated. As opposed to metastatic PC, not every BCR case should require prompt treatment. As a matter of fact, early treatment of low-risk BCR with continuous ADT can produce disadvantages such as an aberration in the natural history of PC with the development of castration resistance before the appearance of metastases (nmCRPC). On the other hand, patients with high-risk BCR (PSA-DT ≤ 9 months) benefit from early local and systemic therapy. For this class of patients, the combination of enzalutamide with leuprolide, either intermittent or continuous, significantly improves MFS when compared to leuprolide alone, maintaining the quality of life unchanged in the asymptomatic phase of the disease.

The introduction of new therapeutic strategies, in particular with ARSIs, has led to a rush towards the concept of anticipation and intensification of therapy in PC. The advantage of this concept is represented by the possibility to block the disease at an earlier stage when the patient’s quality of life is better preserved as well as having a positive impact on overall survival. The major risk is represented by overtreatment in a non-negligible percentage of patients as well as the development of resistance, which can reduce therapeutic options in the subsequent phases. In nmCRPC patients, intensification of therapy with ARSIs is now recommended only in high-risk subjects based on a PSA-DT < 10 months. On the contrary, in mHSPC, the current guidelines recommend the intensification of ADT therapy with ARSIs or chemotherapy in all patients, while performing stratification based on the volume of disease according to the Charteed criteria (low and high volume) or Latitude risk classes (low and high risk).

The EMBARK study represents the first significant evidence in support of the use of an anticipation and intensification of therapy with ARSIs in the BCR phase after primary treatments. In this phase of the disease, the primary advantage of this concept is represented by the delay in the evolution of a metastatic phase (MFS), even with the same OS, maintaining patients in an asymptomatic condition with good quality of life. The greatest risk is that of overtreatment in subjects who could remain non-metastatic for several years even without therapies. As opposed to overt metastatic PC, the possibility of using ARSIs alone in this early disease setting is suggested by the EMBARK study (arm with enzalutamide alone), but with less evidence than with the combination therapy. As more treatment options become available, large-scale analysis based on real-world data must verify the significant results obtained in the context of a phase 3 study.

## Figures and Tables

**Table 1:** Parameters which can contribute in distinguishing BCR after primary treatments into low- and high-risk for early clinical progression. BCR = biochemical recurrence. HRR = homologous recombinant repair. PV = pathogenetic variant.

Parameter	Low-Risk BCR	High-Risk BCR
T stage	T2	T3
ISUP grading	1–2	3–5
Surgical margins	Negative	Positive
Time to primary treatment	>12 months	≤12 months
Pretreatment PSA value	<10 ng/mL	≥10 ng/mL
PSA-DT	≥10 months	<10 months
HRR PV	BRCA2 −	BRCA2 +

**Table 2:** How to define and treat BCR after RP or RT at low and high risk for early progression. BCR = biochemical recurrence. RP = radical prostatectomy. RT= radiotherapy. SRT = salvage radiotherapy. AO = active observation. ADT = androgen deprivation therapy. ARSI = androgen receptor signaling inhibitor. HIFU = high intensity focalized ultrasound.

	BCR after RP		BCR after RT	
	Low Risk	High Risk	Low Risk	High Risk
Definition	PSA-DT ≥ 10 ng/mL	PSA-DT < 10 months	PSA-DT ≥ 10 ng/mL	PSA-DT < 10 months
Imaging to exclude metastases	PSMA PET/CT	PSMA PET/CT	PSMA PET/CT	PSMA PET/CT
Current treatment	- AO - SRT	- SRT - ADT	- AO - Stereotactic RT - Cryoablation - HIFU	ADT
Treatment in the near future	- AO - SRT	- ARSI + ADT	- AO - Stereotactic RT - Cryoablation - HIFU	ARSI + ADT
